# Patient Power: A feasibility study on the impact of providing a bedside notepad to encourage patients to ask questions following surgery

**DOI:** 10.1016/j.pecinn.2024.100257

**Published:** 2024-01-22

**Authors:** Breanna Wright, Justin Aylward, Sharon Allsop, Alyse Lennox, Nicholas Faulkner, Peter Bragge

**Affiliations:** a BehaviourWorks Australia, Monash University, Australia; b Peninsula Health, Australia

**Keywords:** Patient engagement, Patient activation, Patient voice, Communication, Patient safety

## Abstract

**Objective:**

This study aimed to evaluate a behaviour change strategy to enhance the patient voice in the early post-operative setting.

**Methods:**

The Patient Power notepad was evaluated in an uncontrolled, single-group, mixed-methods trial including a patient evaluation survey and staff phone interviews.

**Results:**

Patients thought that the notepad was well-designed and prompted them to think of and ask questions. They strongly agreed that healthcare practitioners answered health-related questions fully and carefully. Staff reported that the notepad not only provided an easy mechanism through which patients and their families could communicate with their healthcare team, but it also created a permissive environment where questions were encouraged.

**Conclusion:**

The Patient Power notepad provided an easy, acceptable and scalable intervention to encourage patients to engage more in their healthcare and specifically to ask questions about their care. By providing a structured tool for capturing patient concerns, symptoms, and questions, this innovation holds the potential to enhance patient satisfaction, treatment adherence, and overall healthcare outcomes.

**Innovation:**

By facilitating comprehensive information exchange and the potential to promote shared decision-making, this innovation has the potential to improve patient satisfaction, treatment adherence, and overall healthcare outcomes.

## Highlights

• A co-designed ‘Patient Power’ notepad was tested in a feasibility study in an Australian public hospital.
• Patients and staff both found the notepad beneficial.
• The ‘Patient Power’ notepad is an easy to use, easy to implement and scalable intervention that can improve communication.

## 1 Introduction

Engaging patients in their care can result in the improvement of health outcomes, reduced healthcare costs and increased patient satisfaction [1]. Thus, emphasis is increasingly being placed on encouraging patients to play a more active role in their health care. This has developed from a growing awareness that many patients are dissatisfied with the information they receive during medical consultations and that there is an association between patient satisfaction and health outcomes [2]. Furthermore, the importance of doctor–patient communication in the process of health care has been well-established, with studies demonstrating clear links between the quality of communication and patient satisfaction, adherence to recommended therapy and clinical outcomes [3].

Patients do not always feel empowered to talk to their healthcare team or to speak up if they have concerns. Even when patients have questions, they may find it difficult to voice them. Barriers to open communication include perceived priorities of staff, how busy staff are, the seriousness of the issue and perceived consequences of speaking up [4]. Some of these barriers, such as perceived busyness of staff, are particularly salient in hospital settings. Inpatient hospital stays involve potentially complicated and confusing inpatient procedures where patients see multiple staff; move locations multiple times; and can experience conflicting information [5]. These processes, combined with the unfamiliar hospital environment, can inhibit active patient engagement in their inpatient healthcare [6].

There may be further disincentives to patients speaking up, including perceived lack of opportunity to ask questions, poor health literacy and confidence, and their perception of constraints on clinicians' time. Inpatient stays typically involve limited engagement with doctors and specialists during a ward round, where the clinical team may only be at the bedside for a short time. Thus, patients may forget questions that they wanted to ask in the moment or perceive that, in the environment, it is unacceptable to ask questions [6]. Affective barriers arise when patients anticipate feeling humiliated, shamed or embarrassed if they ask questions [2].

One way to encourage patients to ask questions when they have them is to provide a clear and easy avenue for them to do so. This may be in the form of question prompt lists which can help patients think of questions to ask or a place to record those questions such as a notebook. Providing patients with appropriately designed and relevant question prompts creates an opportunity for patients to ask questions and encourages them to participate more actively in their care [7]. Facilitated communication through question prompts can also improve the delivery of information by clinicians [7]. Patients who actively participate in their healthcare by asking questions of their doctor are able to control the focus of their interaction and adjust the amount of information provided so that it meets their needs [8].

With greater emphasis placed on patient- and family-centred care at many health services and in the context of limited time and bandwidth to implement complicated interventions, simple strategies are needed that encourage patients and their families to reflect on their concerns and where necessary, express these concerns and ask questions.

The aim of this study was to develop and test a behaviour change strategy to enhance the patient voice in early inpatient post-operative care. This involved the development of a purpose-designed notepad that included question prompts to encourage patients to ask questions and space to record their questions, which their healthcare team could then access and respond verbally as required.

## 2 Methods

### 2.1 Intervention development

The intervention was developed through a structured five-step process. Firstly, an expert panel was convened to steer the project. Second, an evidence review of relevant academic papers was undertaken and synthesised with data from interviews with practice experts. Third, a citizen panel was convened with patients and the public to elicit their perspectives on the topic and potential intervention options. Fourth, a structured stakeholder dialogue was convened to deliberate on the data gathered through the review and consultation activities and prioritise a feasible intervention that could be piloted. Fifth, the intervention plan was refined and co-design workshops were held to develop the intervention materials. Further detail of these steps is provided below.

Expert Panel: A panel of five experts with extensive experience in patient engagement and shared decision-making was convened to steer the focus of the project which was broadly to improve patient engagement in their healthcare and oversee decisions. They refined the research question for the evidence review to ‘*What interventions are effective to improve communication or shared understanding of wound care in the post-operative and discharge contexts’* which informed the next step described below. Following this, the expert panel provided input into recruitment for the stakeholder dialogue and directed the research team to relevant literature.

Evidence Review: A review of relevant academic papers was undertaken using a rapid review methodology [9,10]. The review collected evidence on “interventions to improve communication or shared understanding of wound care in the post-operative and discharge contexts”. A comprehensive search was conducted in the following databases for the years 2014–2018: PubMed, Cochrane Library, Web of Science, Scopus, CINAHL, and the first 50 pages of Google Scholar. The rapid review included three systematic reviews and seven primary studies. The review covered written and verbal communication such as notepads, and inpatient and bedside interventions, such as educational toolkits. The collated evidence was presented at the stakeholder dialogue, described below. In addition to reviewing the academic evidence, two interviews were conducted to inform key implementation challenges; with clinical nurse advisors.

Citizen Panel: A citizen panel of 12 diverse patients, families/carers and members of the general public was convened to elicit their key values and opinions and gain their reflections on possible intervention options. They were presented with a plain language copy of the results from the evidence review to discuss.

Structured Stakeholder Dialogue: A day-long, structured stakeholder dialogue was convened with nine participants representing government, insurance, clinicians, a patient representative, communications and research sectors. Through facilitated discussion, potential interventions were evaluated for their feasibility, sustainability and scalability. This process, identified an intervention to encourage and empower patients to ask questions or raise concerns they had about their care.

The specifics of the intervention were developed through co-design workshops with consumers who had undergone total joint replacement (e.g. hip or knee). This involved workshopping all aspects of a notepad to record questions. The trial design was planned in partnership with the identified trial hospital. A mixed methods approach was selected for the feasibility study to incorporate both qualitative and quantitative insights, allowing for an initial assessment of the notepad.

### 2.2 Intervention implementation

**Fig. 1:**
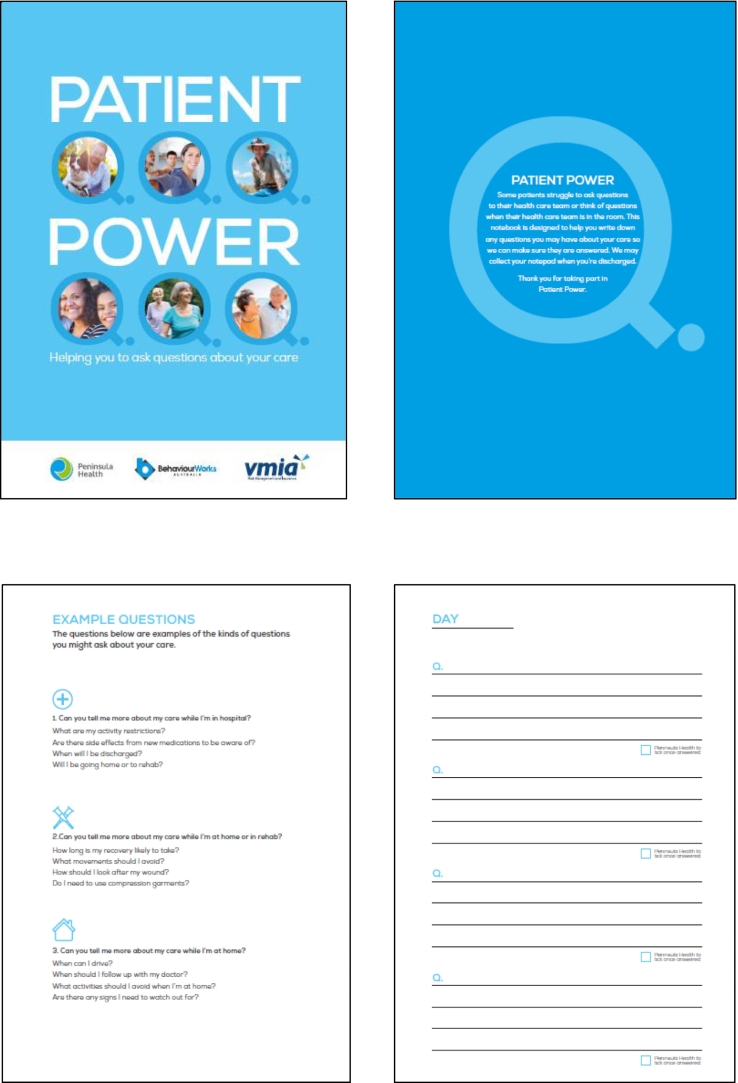
The Patient Power Notepad.

The intervention consisted of a ‘Patient Power’ notepad that was provided to patients at pre-admission for use during their post-operative stay, to facilitate patient voice. The notepad was designed to be placed within reaching distance of the bed, durable (e.g. hard cover), and easy for patients and staff to use. Furthermore, the notepad aimed to help patients record questions when no-one was around so they didn't forget them and removed the pressure to recall questions on the spot. Clinicians were then able to respond when they next saw the patient. The content of the notepad is shown in Fig. 1. Associate Nurse Unit Managers (A/NUMs) were in charge of overseeing the notepad use and ensuring that questions were regularly checked and answered, while encouraging staff to answer questions where they were able to.

As described above, patients, families/carers and members of the general population (who were potential patients and/or family/carers) were included at the formative phase, especially the Citizen Panel, to obtain their views and priorities on intervention options. Following the formative phase of the research, a period of intervention development was undertaken which involved a co-design workshop with patients who had undergone total joint replacement surgery as this population was the focus of the feasibility study. The purpose of the workshop was to co-design the notepad intervention. Patients and members of the public had no further involvement in decisions about the design of the study, or in dissemination of study results.

For patient outcomes, all patients aged 18 or over who underwent hip or knee replacement surgery at Peninsula Health, Victoria, Australia during the trial period were eligible to participate and were provided with the notepad when they were admitted. All eligible patients received the notepad and then had the choice to participate in the study component by completing the survey, which was provided by the nurses, prior to discharge. Patients who were unable to provide consent to participate i.e. Non- English speaking, with cognitive, vision or hearing impairment, were excluded.

For clinician outcomes, all clinicians who worked in the post-operative orthopaedic ward were eligible to participate in a feedback interview at the end of the trial period.

The study aimed to assess the feasibility of the Patient Power notepad intervention using a pragmatic, uncontrolled, single-group, mixed-methods trial. During the five-month trial period, all patients who underwent total joint replacement surgery at the trial hospital were provided with the notepad upon check-in, just prior to surgery and instructed that they could use the notepad for post-operative questions, not pre-operatively. Nurses reminded patients that they could use the notepad to record their questions. Nurses and doctors during their rounds were responsible for checking the notepad and responding to questions. Upon hospital discharge, used pages from the notepad were collected, and patients were invited to complete a survey. At the conclusion of the trial, clinicians in the post-operative orthopaedic ward at the participating hospital were also invited to complete a survey.

As this was a feasibility study, it was not designed nor powered for statistical significance. Nonetheless, the sample size of 20 is consistent with recommendations to include between 12 and 30 participants per arm in feasibility studies [11,12], and with common practices in registered feasibility studies [13].

Three types of data were collected for this study: (1) completed Patient Power notepad pages; (2) patient survey data; and, (3) clinician interview data. To encourage patient participation, the request to complete the survey occurred during the standard discharge procedure. If the patient agreed to participate, the hospital staff member provided the patient with a paper copy of the survey. For the clinician outcomes, phone interviews were scheduled with a purposive sample of staff from the participating ward.

### 2.3 Ethics

This study received ethics approval from the Peninsula Health Human Research Ethics Committee (LNR-47801-PH-2018), and the Monash University Human Research Ethics Committee (18463). It was prospectively registered on the Australian and New Zealand Clinical Trials Registry (ACTRN12618001869268). All participants provided informed consent prior to participating in the trial.

### 2.4 Data collection

Data from patient notepads has not been made publicly available due to the high risk of it containing identifiable information.

Number of questions: The number of questions recorded in each patient's notepad as counted by the researchers.

Types of questions: Two researchers independently conducted a content analysis to categorise the types of questions recorded in the notepads.

Self-reported satisfaction with and perceived usefulness: Self-reported indicators included: the patient experience of using the notepad; their satisfaction and perceptions of whether it was useful; if it encouraged them to ask questions and if they felt that their questions were adequately addressed.

Brief Health Care Climate Questionnaire: This validated questionnaire measures patients' perceptions of the degree to which their clinicians provided supportive communication. The questionnaire contains nine questions; e.g. My healthcare practitioner answers my questions related to my health fully and carefully [14].

Clinician feedback: Clinician feedback on the usefulness and usability of the notepad, including any benefits, drawbacks and workflow implications were elicited during a semi-structured phone interview with a member of the research team.

Quantitative data: Descriptive statistics (means and standard deviations) were reported for all quantitative outcome variables. Negatively framed items were reverse scored.

Qualitative analysis: We used content analysis to identify qualitative themes in patient questions, patients' responses to open-ended survey questions and clinicians' interview transcripts. Two researchers independently coded qualitative data using an inductive analysis approach for the interviews and qualitative survey responses.

## 3 Results

Twenty patients completed the post-intervention evaluation survey, of these twenty, sixteen reported using the notepad. Overall, the majority of indicators were positive. Patients liked having the option to use the notepad during their time in hospital and thought that it was well-designed and prompted them to both think of and ask questions (see Table 1). Patients were satisfied with the care they received during their stay and strongly agreed that patients should ask questions about their care and take an active role in their healthcare (see Table 2, Table 3). Overall, patients felt a lot of trust in their healthcare practitioners and felt able to be open with them about their healthcare. They strongly agreed that healthcare practitioners answered health-related questions fully and carefully. Even patients (*N* = 4) who did not feel the need to the use the notepad were positive about its availability.

**Table 1:** Satisfaction and Usefulness (scale 1–5).

	Mean (SD)
The notepad was useful in communicating with my health care team	4.25 (1.18)
The notepad was useful in prompting me to think of and ask questions	4.42 (1.07)
The notepad was well designed	4.42 (1.07)
The notepad made it easier for me or my family to ask questions	4.35 (1.09)
I like having the option of using the notepad during my time in hospital	4.50 (1.05)

**Table 2:** Confidence (scale 1–5).

	Mean (SD)
I felt confident to ask questions when I thought something wasn't right	4.47 (0.51)
The care I received during my stay in hospital was very good	4.74 (0.45)
I feel confident in the care I received during my stay in hospital	4.74 (0.45)
Doctors or nurses sometimes ignore when I try to ask a question⁎	4.10 (1.29)
Healthcare teams need to listen to patients if they have concerns	4.63 (1.01)
Patients should ask questions about their care	4.60 (1.23)
Patients should take an active role in their healthcare	4.55 (1.23)

⁎ Item has been reverse scored.

**Table 3:** Brief Health Care Climate Questionnaire (scale 1–5).

	Mean (SD)
I feel that my healthcare practitioner has provided me with choices and options about my health	4.59 (0.87)
I feel my healthcare practitioner understands how I see things with respect to my health	4.56 (0.89)
I am able to be open with my healthcare practitioner about my health	4.73 (0.80)
My healthcare practitioner encourages me to ask questions	3.35 (2.43)
I feel a lot of trust in my healthcare practitioner	4.94 (0.25)
My healthcare practitioner answers my questions related to my health fully and carefully	4.75 (0.77)
My healthcare practitioner handles my emotions very well	4.47 (1.06)
I feel that my healthcare practitioner cares about me as a person	4.36 (1.08)
I don't feel very good about the way my healthcare practitioner talks to me about my health*	3.71 (1.68)

### 3.1 Patient feedback

When asked to describe the most useful feature of the notepad, patients appreciated the simplicity and provision of a pen and paper. Positive comments also related to the appropriateness of the sizing and space to record the post-operative day. Patients stated that the notepad was easy to use and acted as a prompt to ask questions without judgement. They liked having the ability to write the questions down so that they did not need to remember them and it helped to ensure that they received answers to their questions. One patient stated that *‘it opened up lines of communication’*, while another commented that the notepad was *‘helpful and very well set out’* and that it was a *‘pleasure to have the chance to use it’.* Although a few patients reported that they did not need to use the notepad, as ‘*staff were proactive’* about answering questions, they could still see they value in having it.

However, in the survey feedback, there was neutral endorsement that “My healthcare practitioner encourages me to ask questions” which suggests that whilst the notepad may have been useful for patients, it did not necessarily act as the same prompt for the healthcare team to engage the patient in asking questions.

### 3.2 Notepad use

The notepad was used 90 times by 16 patients, each distinct question, comment, or entry was considered a separate ‘use’. On average, patients used the notepad 5.6 times during their inpatient stay. While it was mostly used to ask questions (*n* = 56; see Table 4), it was also used as a diary, to make comments and to provide feedback. Of the patients who specified the day on which questions were asked, the notepad was utilised most on Day 1 of their stay. Questions were more likely to be recorded as answered with a tick in the ‘[Hospital] to tick once answered’ box (25/56 questions answered) than diary entries or comments/feedback. However, it is not possible to know how many entries were responded to or acknowledged but not ticked in the notepad. Content analysis of the questions asked in the notepads highlighted the following key themes (see Table 4 for summary of themes and sub-themes); hospital environment, staff, in-hospital processes, decision-making, recovery, and surgical/medical. In particular, there were many questions about how to manage recovery and when to re-start particular activities. There were many instances where patients or their families wanted to clarify or confirm information.

**Table 4:** Notepad question themes.

Question theme	Sub-theme	Example
Hospital environment	Lighting	*Lying on the bed- next to the corridor- why can't you block off the light? Most annoying shining in your eyes all day and part of the night*
	Noise levels	*Why do bells go off constantly?*
Staff	Poor communication	*Physio attended for the first time. I was disappointed at her poor bedside manner. Is it usual to have such poor communication skills and bedside manner?*
In-hospital processes	Washing	*Is it normal for sponge to be done after op (early morning) later in the day? No wash post-op in morning*
	Medical Certificate	*I will need a certificate for work. Can I get one?*
	Wound care	*Does my wound dressing get changed before I leave the hospital? Has dry blood stains.*
	Toileting	*Why do you need to poop before they can take out catheter?*
	Referrals	*How do I get to be referred to pain clinic? Have a further three surgeries and have had chronic pain (3 years now).*
	Follow-up procedures	*What f/up at Frankston hospital over 6 weeks?*
	Other	*Why do I have a neck thing in? I've been told that you don't usually have it but it is in for about 3 days*
Decision-making	Driving	*Who's best to decide when to drive (powers to be)?*
	Rehabilitation	*When does the decision to go to rehab get made?*
	Physiotherapy	*How are milestones over six weeks determined?*
Recovery	Driving	*How soon can I drive a car? I have an automatic.*
	Swelling	*How long until the swelling goes down?*
	Washing	*When can I safely start showering?*
	Alcohol	*How soon can I start drinking alcohol after I return home?*
	Coping at home	*How can I go home if I do not feel I can cope by myself?*
	Pain	*Still pretty sore 2 days after surgery. How long does the severe pain last?*
	Mental health	*Is he feeling mentally ok: anxiety etc?*
	Wound care	*How should I look after my wound? Are there any signs I need to watch out for?*
	Toileting	*When will I be able to use the toilet again, no catheter?*
	Physical activity /Physiotherapy	*What are my activity restrictions at the moment? Physio frequency of home visits?*
	Support contacts	*If I need any help for my health, where I give a ring?*
	Occupational therapy	*Alternative devices for putting on socks (have a sock on off at home, still working it out)?*
Surgical/Medical	Procedure details	*How was my new hip attached?*
	Type of replacement joint	*Did not see doctor prior to surgery to ask what sort of hip replacement I have? Plastic, titanium, ceramic?*
	Drugs	*Withdrawal from codeine, sweaty and nausea. Is it withdrawals or is it from operation?*
	Pain management	*What is the maximum amount of time I can spend in hospital to manage my pain levels?*
	Further procedures	*Do I need another platelet transfusion?*

### 3.3 Staff feedback

Interviews were conducted with six staff who participated in the Patient Power trial, including a doctor, two nurses and three nurse managers, who all had a role in supporting the use of the notepad, and answering questions. The greatest benefits of the Patient Power notepad, as reported by staff, were that it empowered patients to ask questions, acted as a reminder and conveyed permission to speak up, as well as opening up communication channels with family members (although it's unclear if family members used the notepad directly). The notepad also acted as a prompt for staff to encourage patients to ask questions and check in with them, as well as thinking about their care and engaging with discharge planning earlier than they would have done otherwise. Furthermore, the notepad demonstrated that staff cared about their patients, and that patients are valued.

The most useful feature of the notepad from the staff perspective was the design and simplicity. The design was appropriate for its intended use and setting, including the size, spacing, cover design and hard cover. To enhance the notepad, one clinician proposed that it could be supplemented with additional consumer information. Thus, the notepad could potentially become a nexus of information for the patient to keep and refer to. There was support from the staff to use the notepad broadly across health services.

### 3.4 Quality improvement

There were also benefits from a quality improvement perspective. The notepad aligned with Australian National Standards for “Partnering with Consumers” and “Communicating for Safety” [15] and demonstrated commitment to key principles of patient safety and engagement. For example, the notepad made it easier for patients to provide feedback, acting as a *‘direct escalation point’* for staff*.* In one instance, clinicians were able to copy a patient's complaint straight into a complaint form and deliver it. They reported that this was *‘powerful because it was handwritten and that was a direct patient experience’.*

Furthermore, the Patient Power notepad did not have any negative impacts on workflow and there were no reported drawbacks to using the notepad. The only comments on workflow raised by staff were in relation to trial processes (e.g. collecting forms and distributing surveys).

## 4 Discussion and conclusion

### 4.1 Discussion

The Patient Power notepad was readily endorsed by both patients and staff, with both groups reporting that it achieved its stated aim of encouraging patients to ask more questions. Additional benefits included creating a permissive environment and sending the message that patients are valued and that their voice is important. No drawbacks to using the notepad were identified by patients or staff.

#### 4.1.1 Usability

The notepads provided an easy intervention that patients and staff readily endorsed. By providing an easy to implement notepad, a pressure-free communication channel was opened up between patients, families and their healthcare team. This allowed patients and their families to ask questions about the things that they needed more information on or worried about. This is aligned with previous research that indicates providing patient- oriented and simple tools to enable patient engagement is effective [16].

Patients reported being very positive about the option of having the notepad available during their stay in hospital and felt that it helped both them and their families to ask questions. Patients also strongly agreed that they should take an active role in their healthcare and ask questions.

Even patients who did not feel the need to the use the notepad were positive about its availability. This is in line with previous research that found only around 50% of patients use question prompt lists if offered one [7]. Previous research has found that standalone question prompts are not effective in isolation [2] and indeed some have concluded that question prompts alone cannot enhance communication between patients and staff [7]. The Patient Power notepad combined question prompts with a dedicated space to record questions. Additionally, in this study, some of the patients had undergone a total joint replacement prior to their current admission, which may have contributed to them having fewer questions as they had already been through the process and the hospital experience. The themes developed from the questions asked in the notepads indicate that while there is substantial breadth in the types of questions asked they do center around patients wanting to understand their immediate circumstances, including in-hospital processes, and recovery processes. This is similar to previous research that has found that patients who underwent joint replacement surgery primarily had questions about their immediate recovery [17,18]. However, our findings extend previous research by revealing that patients also have questions about their immediate in-hospital experience including queries about the hospital environment, staff and self-care.

Qualitative feedback from patients indicated that the notepad achieved its goal of overcoming some of the identified barriers to patients asking questions. In particular, patients reported that it helped them ask questions because they did not have to remember the questions at a particular time when the clinician was present and removed any feelings of judgement associated with asking questions. The notepad itself also acted as a prompt and reminder for patients to ask questions. Importantly, patients also reported that this simple intervention helped to open up lines of communication with their healthcare team.

Staff also reported that the notepad did not disrupt workflows and helped enable communication channels between patients and staff. Furthermore, the notepad could help staff and the health service meet Australian national standards; in particular “Partnering with Consumers” and “Communicating for Safety” [15].

The notepad was originally designed to encourage patients to ask questions, however, it is clear from the responses of patients and staff that the notepads did more than this. It encouraged patients to think and engage in their care, it opened lines of communication with the healthcare team, which patients repeatedly report as being difficult [3], and it set an expectation that asking questions and speaking up is normal in the hospital setting. The patient findings on the notepad are similar to other patient notepads such as ‘Dear Doctor’ which similarly demonstrated improved patient satisfaction [8].

#### 4.1.2 Scalability

The Patient Power notepad provided an easy and reasonably cheap intervention that could be easily scaled across appropriate settings and populations. While the Patient Power notepad was specifically co-designed with patients and healthcare professionals, it may be that providing any simple device to record questions would serve the same purpose. However, staff did report that the bespoke design of the notepad contributed to its success, including both the functional aspects such as spacing on the page and having a hardcover, as well as the message that the purpose-built design sent to patients facilitating patient voice. There was a perception that a generic notepad would not achieve the same effect.

*E*-devices could also achieve similar goals to the Patient Power notepad. These could address linguistic diversity as well as other inclusive aspects such as allowing patients to audio-record their questions (which could assist in accessibility for visually impaired patients). There may also be barriers related to literacy and health literacy. Encouraging family and carers to engage could help overcome this to some extent. The Patient Power notepad was designed with question prompts to help patients think of the types of questions they may have which may help with health literacy, although this requires further exploration. Adapting patient tools so that they meet patient needs is a key part of patient-centred care.

#### 4.1.3 Strengths and limitations

This study provides evidence for the feasibility and usability of providing patients with notepads to enhance communication and encourage greater engagement with their care. However, some limitations should be acknowledged. Firstly, this was a non-controlled feasibility study, which means that there is no comparative data by which to judge the effectiveness of the notepad, the feasibility study means that strong conclusions cannot be drawn. Secondly, as a feasibility study, the population sample was relatively small, which may affect the generalisability of the results. Furthermore, there were challenges in ensuring that all patients who had received a notepad were given surveys, as some patients were discharged before the survey could be provided and collected by the research team and there may have been a response bias in that those who used the notepad or were more positive towards it were more likely to complete the evaluation survey, however we do not have the data to know if this occurred. It was reported by staff that many more patients used the notepads, but their surveys were not able to be collected. Due to the design of the feasibility study, we weren't able to include family or carers in the evaluation, however this would be useful to explore in further research. Furthermore, staff interviews were purposefully sampled, and unlikely to be truly representative of the workforce, a more comprehensive evaluation of all staff is required.

### 4.2 Innovation

This innovative tool addresses many identified challenges of effective patient-provider interactions by providing a structured notepad for capturing vital information during the post-operative period. The co-designed notepad incorporates features to facilitate communication between patients and health care providers, it is durable, easy to use and inclusion of question prompts helps to address known barriers to patients asking questions. This innovation promotes a patient-centered approach, enabling healthcare providers to capture and address patient needs more comprehensively. By facilitating comprehensive information exchange and the potential to promote shared decision-making, this innovation has the potential to improve patient satisfaction, treatment adherence, and overall healthcare outcomes.

Whilst innovation often evokes technological advancements, sometimes simple solutions are just as innovative. This study demonstrates the promise of a simple and straight-forward intervention. Embedding co-design in the development of interventions may facilitate finding simple solutions that consider patients' needs and preferences.

### 4.3 Conclusion

Overall, the Patient Power notepad was an easy, acceptable and scalable intervention to encourage patients to engage more in their healthcare and specifically to ask questions about their care. Both patients and staff reported multiple benefits of having the Patient Power notepad available and using it as a communication tool with their healthcare team. There were no recorded drawbacks of having the notepad.

## Funding statement

This research was funded by the Victorian Managed Insurance Authority (VMIA) (Ref: VMIA-MIRI 2016).

## CRediT authorship contribution statement

**Breanna Wright:** Writing – review & editing, Writing – original draft, Supervision, Project administration, Methodology, Conceptualization. **Justin Aylward:** Writing – review & editing, Supervision, Project administration. **Sharon Allsop:** Writing – review & editing, Supervision, Project administration. **Alyse Lennox:** Writing – review & editing, Writing – original draft, Project administration, Methodology, Formal analysis. **Nicholas Faulkner:** Writing – review & editing, Methodology, Formal analysis. **Peter Bragge:** Writing – review & editing, Methodology, Funding acquisition, Conceptualization.

## Declaration of competing interest

The authors declare no conflicts of interest.

## Appendix A Supplementary data

Supplementary Material Image 1
