# Supplementary material for: Patient Power: A feasibility study on the impact of providing a bedside notepad to encourage patients to ask questions following surgery
Source: PEC Innov. 2024 Jan 22;4:100257. doi: 10.1016/j.pecinn.2024.100257 (PMC10839754; doi:10.1016/j.pecinn.2024.100257)
Supplement: Supplementary file 1 — Supplementary Material [file mmc1.docx]

# Supplemental Material

## Patient Survey

**Patient Power Project**

Patient Feedback Survey

During your stay at Peninsula Health, you were provided with a ‘Patient Power’ paper and pen notepad to record any questions you had about your care and help you communicate with your health care team. Before you leave, we’d like to ask for your feedback on the notepad and your experiences.

Please indicate the extent to which you agree with the statements below

|  | Strongly disagree | Somewhat disagree | Neither agree nor disagree | Somewhat agree | Strongly agree |
| --- | --- | --- | --- | --- | --- |
| The notepad was useful in communicating with my healthcare team |  |  |  |  |  |
| The notepad was useful in prompting me to think of and ask questions |  |  |  |  |  |
| The notepad was well designed |  |  |  |  |  |
| The notepad made it easier for me or my family to ask questions |  |  |  |  |  |
| I liked having the option of using the notepad during my time in hospital |  |  |  |  |  |

|  | Strongly disagree | Somewhat disagree | Neither agree nor disagree | Somewhat agree | Strongly agree |
| --- | --- | --- | --- | --- | --- |
| I felt confident to ask questions when I thought something wasn’t right |  |  |  |  |  |
| The care I received during my stay in hospital was very good |  |  |  |  |  |
| I feel confident in the care I received during my stay in hospital |  |  |  |  |  |

|  | Strongly disagree | Somewhat disagree | Neither agree nor disagree | Somewhat agree | Strongly agree |
| --- | --- | --- | --- | --- | --- |
| Doctors or nurses sometimes ignore when I try to ask a question |  |  |  |  |  |
| Healthcare teams need to listen to patients if they have concerns |  |  |  |  |  |
| Patients should ask questions about their care |  |  |  |  |  |
| Patients should take an active role in their healthcare |  |  |  |  |  |

Is there anything you would change about the notepad or its use?

_____________________________________________________________________________________________________________________________________________________________________________________________________________________________________________________________________________________________________________________________________________________________________________

What, if any, was the most useful feature of the notepad?

_____________________________________________________________________________________________________________________________________________________________________________________________________________________________________________________________________________________________________________________________________________________________________________

We now have a few questions about your relationship and interactions with your healthcare practitioners. Practitioners have different styles in dealing with patients. Your responses will be confidential.

|  | Strongly disagree | Somewhat disagree | Neither agree nor disagree | Somewhat agree | Strongly agree |
| --- | --- | --- | --- | --- | --- |
| I feel that my healthcare practitioner has provided me with choices and options about my health |  |  |  |  |  |
| I feel my healthcare practitioner understands how I see things with respect to my health |  |  |  |  |  |
| I am able to be open with my healthcare practitioner about my health |  |  |  |  |  |
| My healthcare practitioner encourages me to ask questions |  |  |  |  |  |
| I feel a lot of trust in my healthcare practitioner |  |  |  |  |  |
| My healthcare practitioner answers my questions related to my health fully and carefully |  |  |  |  |  |
| My healthcare practitioner handles my emotions very well |  |  |  |  |  |
| I feel that my healthcare practitioner cares about me as a person |  |  |  |  |  |
| I don’t feel very good about the way my healthcare practitioner talks to me about my health |  |  |  |  |  |

## Clinician Feedback Survey

You are invited to participate in an evaluation survey for the Patient Power project which was trialled on the Port Phillip Ward in partnership with Monash University. The purpose of this research was to investigate the usefulness and feasibility of providing patients with notepads to help them ask questions about their care.

This survey is being sent to all staff who were involved in the trial or worked on Port Phillip Ward during the trial. It should take approximately 5 minutes to complete.

By completing this survey you will be providing information to evaluate the trial and that may assist in improving the notepad intervention for future iterations.

This survey is anonymous, your name is not recorded anywhere on the survey.

If you wish to take part in this research project please complete the survey. By completing the survey you are telling us that you

- Understand what you have read
- Consent to taking part in the research project
- Consent to the use of your survey data as described

Consent in this survey is voluntary, if you do not wish to take part you do not have to.

Once you have submitted your responses they remain in the data collected by the survey.  If you decide that you would like to withdraw from participation after you have submitted your answers your data cannot be removed as it was not identified.

Results of this survey will be available on request from Port Phillip Ward and will be submitted for publication and presented at conferences and seminars.

The ethical aspects of this project have been approved by the Peninsula Health Human Research Ethics Committee.

If you have any complaints about any aspect of this research project, the way it is being conducted or any questions about being a research participant in general you may contact:

The Manager Research Program Peninsula Health

Telephone**:** 9788 1473

E-mail: researchethics@phcn.vic.gov.au

Please ensure that you print this section or retain a copy for your records.

To evaluate the usefulness and transferability of the Patient Power notepad intervention, we would appreciate your feedback on the trial.

**During the Patient Power project:**

Did the Patient Power notepad help you communicate with your patients?

_____________________________________________________________________________________________________________________________________________________________________________________________________________________________________________________________________________________________________________________________________________________________________________

Does the notepad fit with work flows?

_____________________________________________________________________________________________________________________________________________________________________________________________________________________________________________________________________________________________________________________________________________________________________________

What, if any, was the greatest benefit of the Patient Power notepad?

_____________________________________________________________________________________________________________________________________________________________________________________________________________________________________________________________________________________________________________________________________________________________________________

Is there anything you would change about the notepad or its use?

_____________________________________________________________________________________________________________________________________________________________________________________________________________________________________________________________________________________________________________________________________________________________________________

Were there any difficulties in using the notepads from the health care practitioner perspective?

_____________________________________________________________________________________________________________________________________________________________________________________________________________________________________________________________________________________________________________________________________________________________________________
